# Ultrasonically Processed WSe_2_ Nanosheets Blended Bulk Heterojunction Active Layer for High-Performance Polymer Solar Cells and X-ray Detectors

**DOI:** 10.3390/ma14123206

**Published:** 2021-06-10

**Authors:** Hailiang Liu, Sajjad Hussain, Jehoon Lee, Dhanasekaran Vikraman, Jungwon Kang

**Affiliations:** 1 Department of Electronics and Electrical Engineering, Dankook University, Yongin 16890, Korea; liuhailiang107@gmail.com (H.L.); usyj0512@gmail.com (J.L.); 2 Institute of Nano and Advanced Materials Engineering, Sejong University, Seoul 05006, Korea; shussainawan@gmail.com; 3 Division of Electronics and Electrical Engineering, Dongguk University-Seoul, Seoul 04620, Korea

**Keywords:** WSe_2_ nanosheets, charge transport, mobility, polymer solar cell, sensitivity

## Abstract

Two-dimensional (2D) tungsten diselenide (WSe_2_) has attracted considerable attention in the field of photovoltaic devices owing to its excellent structure and photoelectric properties, such as ordered 2D network structure, high electrical conductivity, and high mobility. For this test, we firstly prepared different sizes (NS1–NS3) of WSe_2_ nanosheets (NSs) through the ultrasonication method and characterized their structures using the field emission scanning electron microscope (FE-SEM), Raman spectroscopy, and X-ray powder diffraction. Moreover, we investigated the photovoltaic performance of polymer solar cells based on 5,7-Bis(2-ethylhexyl)benzo[1,2-c:4,5-c′]dithiophene-4,8-dione(PBDB-T):(6,6)-phenyl-C71 butyric acid methyl ester (PCBM) with different WSe_2_ NSs as the active layer. The fabricated PBDB-T:PCBM active layer with the addition of NS2 WSe_2_ NSs (1.5 wt%) exhibited an improved power conversion efficiency (PCE) of 9.2%, which is higher than the pure and NS1 and NS3 WSe_2_ blended active layer-encompassing devices. The improved PCE is attributed to the synergic enhancement of exciton dissociation and an improvement in the charge mobility through the modified active layer for polymer solar cells. Furthermore, the highest sensitivity of 2.97 mA/Gy·cm^2^ was achieved for the NS2 WSe_2_ NSs blended active layer detected by X-ray exposure over the pure polymer, and with the NS1 and NS2 WSe_2_ blended active layer. These results led to the use of transition metal dichalcogenide materials in polymer solar cells and X-ray detectors.

## 1. Introduction

Optoelectronic devices based on organic semiconductors have received considerable attention in recent times [1]. Currently, organic semiconducting materials have been considered for a wide range of applications, such as organic solar cells [2,3,4], organic light-emitting diodes [5,6], sensors [7,8] and photodetectors [9,10]. This is mainly due to the excellent properties of organic semiconductor materials, such as their low cost, lightweight design, flexibility, and excellent thermal and mechanical stability [11,12,13,14,15,16]. A wide-bandgap polymer, 5,7-Bis(2-ethylhexyl)benzo[1,2-c:4,5-c′]dithiophene-4,8-dione (PBDB-T) is one of the most recognized and effective donor materials in polymer solar cells (PSC). Moreover, most organic-based semiconducting devices use a fullerene derivative phenyl-C70-butyric acid methyl ester (PCBM) as an acceptor due to its high rate of conductivity, which is combined with the photon charge conversion layer (active layer) [17,18,19,20,21]. Among the donor materials, PBDB-T-conjugated polymers are the most studied and well-recognized choice of material due to their unique properties, such as semicrystalline structure and high hole/electron mobility, as well as their ease of interaction with fullerene-based acceptors such as PCBM [22]. PBDB-T exhibits an excellent absorption coefficient and is located at the deepest level of the occupied molecular orbital (HOMO) [23,24]. For this reason, PBDB-T:PCBM is considered as the driving force for further in-depth research on organic solar cells, sensors and photodetectors.

Third-generation bulk heterojunction (BHJ) PSCs are cheaper and weigh less compared to first-generation single-crystal silicon-based solar cells and second-generation compound semiconductor-based solar cells [25,26,27,28]. However, their power conversion efficiency (PCE) is insufficient compared to that of other silicon-based devices. This is mainly due to the low mobility of polymer semiconductors, which leads to poor charge transportability [29]. The intrinsic potential of the active layer film and the number of photogenerated carriers generated by the recombination effect are limited by their thickness, which makes it difficult to achieve higher PCE. It is still a considerable challenge to fabricate high-performance BHJ organic solar cells with superior performance. Recently, ternary hybrid-based solar cell research works have attracted growing attention; for example, Sygletou et al. [30] demonstrated that solar cell PCE was improved by 12.5% through the doping of WS_2_–AuNSs into the active layer (PCDTBT:PCBM). Elsewhere, Wu et al. [31] achieved an improved PCE by using different contents of graphene quantum dots (GQDs) for doping in the active layer of P3HT:PCBM. Finally, Ahmad et al. [32] successfully manufactured the ternary hybrid-based active layer of P3HT:PCBM:MoS_2_ NSs with a PCE increment of 32.71% when compared to the performance of an active layer-encompassing device without MoS_2_ added. The PCE enhancements in the ternary hybrid layers are mainly due to their enriched capacity for light absorption and their harmonizing absorption of solar radiation [33,34]. In this respect, interest in ternary-based research has grown recently in order to improve the photovoltaic performance of PSCs.

X-rays have been widely used in the detection of indirect/direct methods and have a wide range of application prospects, including industrial inspection, scientific research (crystallography) and in the field of medicine [35,36,37,38,39]. The coupling of an indirect photodetector and a CsI (T1) scintillator is a common detection method in which the scintillator converts incidental X-rays into visible light. The visible light is then absorbed by the active layer, thereby forming an electron–hole pair to excite charge carriers [21,40]. In a recent report, an X-ray detector based on a ternary system was studied. Unlike the traditional hybrid device concept, a device based on a ternary structure can use the interaction between the organic semiconductor and the additive material to generate surplus-free carrier selection, thus creating a highly sensitive detector. For example, Thirimane et al. [41] reported a ternary hybrid detector based on an organic BHJ-bismuth oxide composite material with a sensitivity of 1712 μCmGy^−1^·cm^−3^ under 50 kV soft X-rays. The organic semiconductors can be manufactured at low cost, at room temperature (over a large area of flexible/wearable substrate), and with an adaptable methodology for complex structures, which will more easily meet the requirements of commercialization.

Transition metal dichalcogenides (TMDs) are attractive semiconductors used in various electronics and optoelectronics [42,43]. TMDs are mainly composed of sandwiched metal (M = Mo, W, etc.) atoms between chalcogenide atoms (such as S, Se, or Te). They possess a unique chemical composition and unique physical properties, such as a high capacity for light absorption, a high carrier mobility, and high bandgap tunability, making them promising as complementary light absorbers and as additional-charge transport materials for high-efficiency ternary devices [44,45,46,47]. Usually, the BHJ structure is composed of a p-type polymer donor and an n-type fullerene acceptor material [48,49]. When the additives are introduced between the polymer donor and the acceptor, it enhances the exciton dissociation and charge transfer activities through interfacing characteristics. An inter-penetration of the nanostructure’s transport network in the active layer can significantly improve carrier mobility. However, the key point of device performance is based on the involvement and interaction of a third component with the thin BHJ layer, which includes uncontrollable factors such as shape and size. For this reason, it is a challenging step to produce suitable nanomaterials with compatible morphological properties in order to maximize the performance of ternary devices.

In this work, we used a simple and convenient method to tune the sizes of WSe_2_ NSs by ultrasonication, and then added different concentrations of WSe_2_ NSs to the polymer heteroatoms active layer of PBDB-T:PCBM to improve its inherent attributes. The experimental results showed that the NS2 WSe_2_ NSs-suspended active layers (1.5 wt%) produced an improved PCE of 9.2%, which increased by 13.5% compared to the pristine polymer junction device PCE of 8.1%. In addition, under the exposure of X-rays, the NS2 WSe_2_ NSs-incorporated (1.5 wt%) PBDB-T:PCBM active layer obtains a sensitivity level of 2.97 mA/Gy·cm^2^.

## 2. Experimental Section

### 2.1. Preparation of WSe_2_ Nanosheets

Firstly, 1 g of WSe_2_ commercial powder was mixed with 100 mL of ethanol solution. The solution was then subjected to sonication at different times, such as 6, 12 and 18 h in a sonic bath under 60 W. Next, it was centrifuged at 8000 RPM for 5 min to retain the precipitate. The collected precipitate was then kept in a vacuum-heating oven until the ethanol evaporated. Finally, the prepared WSe_2_ nanosheets were used for device fabrication and characterization. Based on the sonication times of 6, 12 and 18 h for WSe_2_ preparation, the final products are named “NS1”, “NS2”, and “NS3”, respectively, in the following text. The WSe_2_ nanosheets’ ultrasonic preparation parameters are listed in Table S1 (Supplementary Materials).

### 2.2. Device Fabrication

**Figure 1:**
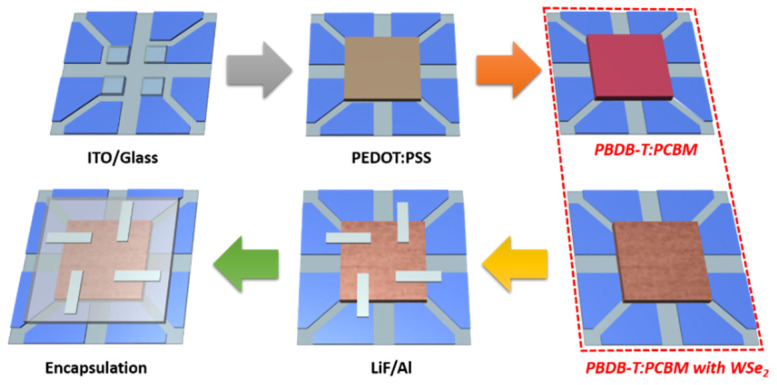
Schematic process for the fabrication of pure and WSe_2_ NSs-suspended PBDB-T:PCBM active layer comprising a BHJ device.

The indium tin oxide (ITO)-patterned glass substrates were cleaned sequentially with acetone, methanol, and isopropyl alcohol for 5 min by sonication treatment, then dried in a vacuum oven at 100 °C, before finally being exposed to UVO treatment for 15 min. The PEDOT:PSS was spin-coated onto the cleaned ITO substrate at 3000 rpm for 30 s and then annealed at 150 °C for 30 min, resulting in a 40 nm thick PEDOT:PSS. For the pure active layer formation, a mixture of PBDB-T and PCBM (with a weight ratio of 2:3) was completely dissolved in chlorobenzene with a concentration of 20 mg/mL, then subjected to constant stirring for 3 h at 60 °C. Next, the prepared solution was spin-coated onto the PEDOT:PSS layer at 1100 rpm for 30 s, and treated by thermal annealing at 150 °C for 10 min. To prepare the WSe_2_ NSs blended active layer, the selective concentration (1, 1.5, and 2 wt%) of prepared WSe_2_ NSs (NS1–NS3) was dissolved in isopropyl alcohol and then mixed with the chlorobenzene mixture (PBDB-T:PCBM—2:3 ratio), before being subjected to constant stirring for 3 h at 60 °C. The WSe_2_ NSs blended PBDB-T:PCBM active layer was then spin-coated onto the PEDOT:PSS layer at 1100 rpm for 30 s, and treated by thermal annealing at 150 °C for 10 min (Table S2). Finally, a 5 nm lithium fluoride (LiF) layer and a 120 nm Al cathode were deposited on top of the active layer by thermal evaporation under a pressurized environment of 3 × 10^−7^ torr. The fabricated device contains four cell structures with an active area of 0.04 cm^2^. To prevent the exposure of the fabricated detector to oxygen and humidity, it was enclosed under a glass lid in a glove box. The device fabrication scheme of a patterned ITO substrate with different active layers (PBDB-T:PCBM (92 nm) and PBDB-T:PCBM:WSe_2_ (89 nm)) is shown in Figure 1.

### 2.3. Characterization

Field emission scanning electron microscopy (FE-SEM, Hitachi S-4700, Tokyo, Japan) was used to describe the morphology and sizes of WSe_2_ NSs. Raman measurements were carried out using a Renishaw inVia (RE04, Gloucestershire, UK) spectrometer with a laser wavelength of 532 nm and an incident power of 5 mW for WSe_2_ NSs. The WSe_2_ NSs structural characteristics were characterized using in-plane X-ray diffraction (XRD, Rigaku D/Max-2500, Tokyo, Japan) with Cu-Kα radiation operated at 50 kV and 300 mA. Light absorption spectra were obtained using a UV–vis spectrophotometer (Optizen 2120UV, K LAB, Daejeon, Korea) for the pure and WSe_2_ NSs blended PBDB-T: PCBM active layers. Atomic force microscopy (AFM) measurements were obtained for the prepared active layers using Park Systems, XE-150 (Suwon, Korea), with a non-contact operating mode. The current density–voltage (J–V) characteristics of PSCs were measured with an electrometer (Keithley 6571B, Tektronix, Inc., Beaverton, OR, USA) under the exposure of an AM 1.5G-filtered Xe lamp with an intensity of 100 mW/cm^2^.

The X-ray detector combined with the scintillator was characterized under X-ray exposure. The emission spectrum of the CsI(Tl) scintillator (Hamamatsu Photonics J1311, Shizuoka, Japan) was measured under X-ray irradiation with a spectrometer (AvaSpec ULS2048L, StellarNet, Inc., Tampa, FL, USA). The prepared detector was placed at a distance of 30 cm from the X-ray generator, with the operation of the X-ray generator fixed under the conditions of 80 kVp and 60 mA·s, before being irradiated for 1.57 s. In order to collect the charge under the X-ray exposure period, a bias voltage of 0.6 V between the cathode and anode of the detector was applied. In addition, at the same distance (30 cm), the X-ray exposure dose was measured using an ion chamber (Capintec CII50, Mirion Technologies (Capintec), Inc., Florham Park, NJ, USA), and the absorbed dose from exposure to the X-ray was 3.14 mGy. The radiation parameters were calculated using the following Formulas (1) and (2), which correspond to the collected current density (CCD) under X-ray irradiation conditions and the dark current density (DCD) under X-ray irradiation conditions, respectively. The sensitivity of the X-ray detector was calculated using a Formula (3), which indicates that the current generated is proportional to the absorbed dose.

(1) CCD [μAcm2]=Collected Current during X-ray ONExposed Detection Area

(2) DCD [μAcm2]=Collected Current during X-ray OFFExposed Detection Area

(3) Sensitivity [μAmGy·cm2]=CCD−DCDAbsorbed Dose

## 3. Results and Discussion

**Figure 2:**
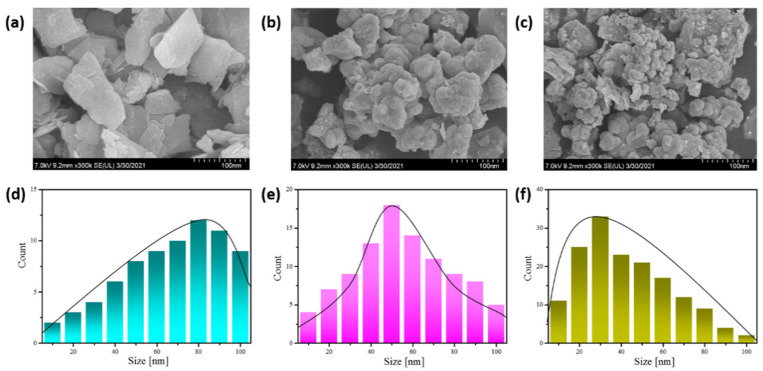
SEM images of WSe_2_ NSs (**a**) NS1 (6 h sonication), (**b**) NS2 (12 h sonication), and (**c**) NS3 (18 h sonication); (**d**–**f**) particle size distribution of WSe_2_ NSs (**d**) NS1 (6 h sonication), (**e**) NS2 (12 h sonication) and (**f**) NS3 (18 h sonication).

The morphological characteristics of different WSe_2_ NSs were evaluated by FE-SEM measurements. Figure 2a–c show the FE-SEM images of NS1, NS2 and NS3 WSe_2_, respectively. The prepared NS1 WSe_2_ produces the agglomerated larger-size granular structure with inhomogeneous shapes and sizes. Moreover, the observed surface reveals the voids and hillocks of a natured morphology. The sizes of the grains were estimated using line profiling with FE-SEM. The line profile of Figure 2d reveals NS1 WSe_2_ NSs with an average size of ~80 nm. Agglomerated grain bunches made of nano-sized grains are observed for NS2 WSe_2_ (Figure 2b). Furthermore, a line profile (Figure 2e) of NS2 WSe_2_ explores the ~50 nm diameter of average grain sizes. For NS3 WSe_2_, the fragmented sizes of differently shaped grains are shown in Figure 2c and its line profile (Figure 2f) indicates minimized sizes of grains with a ~30 nm average diameter. The observed results prove that the sizes of WSe_2_ NSs grains are purely affected by the time of ultrasonic treatment due to the dispersion of nanosheets during the ultrasonic treatment [50].

**Figure 3:**
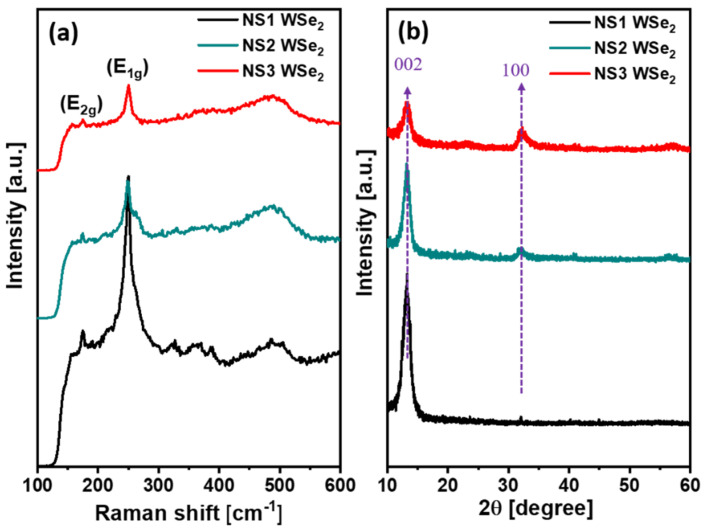
(**a**) Raman spectra and (**b**) XRD patterns of NS1–NS3 WSe_2_ NSs.

The structural property of NS1–NS3 WSe_2_ was characterized using Raman scattering analysis. Figure 3a shows the Raman scattering profiles of WSe_2_ NSs. The Raman scattering of NS1 displays two distinct characteristic peaks of E_2g_ mode and E_1g_ mode, located at 173.99 cm^−1^ and 250.22 cm^−1^, respectively [51,52]. For the NS2 and NS3 WSe_2_ nanostructures, the peak positions are kept constant but their peak intensities are considerably altered. In our observation, the Raman peak intensities reduced considerably after an increase in ultrasonic treatment. The layered structure of WSe_2_ bonded with weak Van der Waals forces between their layers. During the longer ultrasonic bath, weak forces collapsed and induced the agglomeration of the bulk nature of WSe_2_ [53]. In addition, due to the dispersion of the layered structure and nano-sheet sizes, the Raman phonon modes either significantly broadened or were strongly suppressed [52]. These agglomeration characteristics and size decrements are clearly portrayed in the Raman signals. The crystalline nature of NS1–NS3 WSe_2_ was characterized by XRD analysis. Figure 3b reveals the diffraction peaks of WSe_2_ located at 13.28° and 32.05° corresponding to the (002) and (100) lattice planes, respectively. The (002) peak intensity is significantly reduced, while the (100) plane intensity is considerably greater with an increase in sonication time [54,55]. The d-spacing of the (002) peak ascribes the single layer thickness of WSe_2_. After the lengthiest sonication of 18 h, the reduction in (002) peak intensity suggests the destacking/termination of the WSe_2_ layered architecture. The observed results are consistent with the Raman observation. However, unrelated layers randomly folded between other layers, resulting in an increase in (100) lattice-plane peak intensity. The observed results decoded the role of sonication time to produce the highly active WSe_2_ NSs.

**Figure 4:**
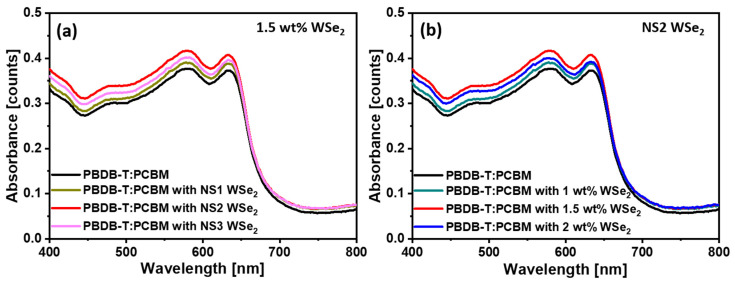
(**a**) UV–vis absorption spectra of pure and NS1–NS3 WSe_2_ NSs blended PBDB-T:PCBM film; (**b**) UV–vis absorption spectra of pure and different concentrations of NS2 WSe_2_ blended PBDB-T:PCBM film.

The effect of incorporation of different WSe_2_ NSs with the PBDB-T:PCBM active layer on the optical properties was investigated by ultraviolet–visible absorption spectroscopy. Figure 4a shows the absorption spectra of pure and NS1–NS3 WSe_2_ NSs blended PBDB-T:PCBM film. The pristine PBDB-T:PCBM film’s absorption spectrum displays several characteristic features with the three distinct peaks at 474, 580 and 632 nm. An observed weak peak centered at 474 nm is associated with the absorption following the extended conjugation of PCBM in the solid state, and the doublets at 580 and 632 nm attribute to the interchain vibrational absorption of ordered PBDB-T chains. Compared to the pristine PBDB-T:PCBM, the PBDB-T:PCBM:WSe_2_ hybrid displays an enhancement in the absorption profile for different NS1–NS3 WSe_2_ (1.5 wt%), as shown in Figure 4a. Furthermore, the different concentrations, such as 1, 1.5 and 2 wt%, of NS2 WSe_2_ NSs blended PBDB-T:PCBM films’ absorption profiles are shown in Figure 4b, which depicts a high absorption behavior for the 1.5 wt% NS2 WSe_2_. The addition of WSe_2_ NSs with the PBDB-T:PCBM active layer provides a superior photon transmission path and enhances photon absorption characteristics.

**Figure 5:**
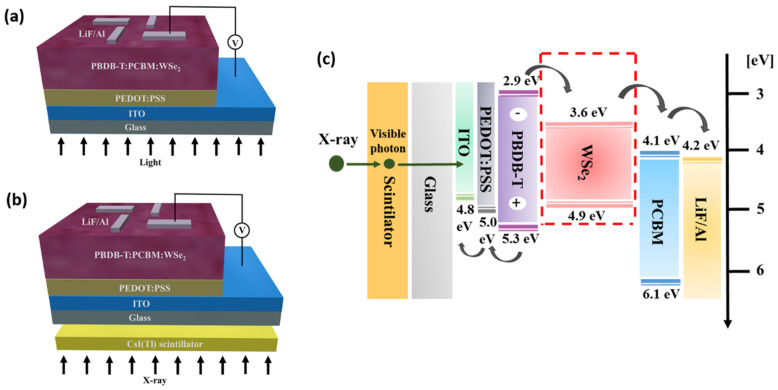
Schematic illustration of (**a**) hybrid polymer solar cell structure; (**b**) X-ray detector structure combined with ScI(Tl) scintillator; (**c**) energy level diagram of the device.

The impact of the incorporation of WSe_2_ NSs with an active layer on the photovoltaic and photodetector performances of ITO/PEDOT:PSS/PBDB-T:PCBM:WSe_2_/LiF/Al device was measured by current density–voltage (J–V) characteristics. The schematic of the hybrid polymer solar cell’s structure and the X-ray detector’s structure is shown in Figure 5a,b, respectively. Figure 5c shows the energy level diagram of each component used for the fabrication of the device. For the X-ray detector, the CsI(Tl) scintillator was constructed, which consisted of 400 μm thick CsI and 0.5 mm thick Al. The induced X-ray photons were converted through the scintillator, and were then absorbed by the active layer (PBDB-T:PCBM:WSe_2_ NSs) to create electron–hole pairs. The transfer of electrons/holes through the cathode/anode thus collected charges. According to the energy band position, additive WSe_2_ NSs help the movement of electrons from PCBM to the cathode, whereas the hole transport layer (HTL) of PEDOT:PSS helps the movement of holes towards the anode.

**Figure 6:**
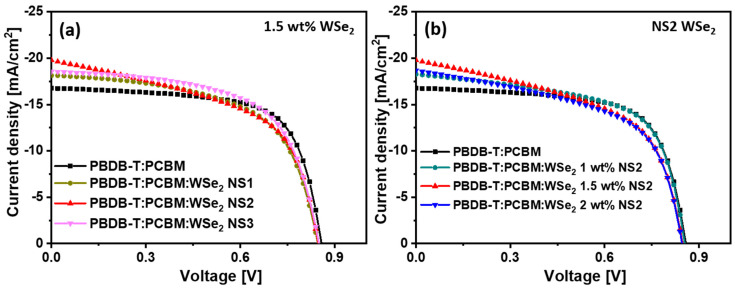
J–V characteristics: (**a**) pristine and different WSe_2_ NSs blended PBDB-T:PCBM active layers; (**b**) pristine and different amounts of NS2 WSe_2_ NSs blended into the PBDB-T:PCBM active layer.

To explore the photovoltaic behavior, the J–V characteristics of pristine PBDB-T:PCBM and WSe_2_ NSs-incorporated PBDB-T:PCBM devices were measured under AM 1.5 G conditions at an illumination intensity of 100 mW/cm^2^. Figure 6a shows the J–V characteristics of devices comprising pure and NS1–NS3 WSe_2_ NSs (1.5 wt%)-incorporated PBDB-T:PCBM active layers. Using a PSC device, we showed that the pristine PBDB-T:PCBM active layer displayed a short-circuit current density (Jsc) of 16.81 mA·cm^2^ and an open-circuit voltage (Voc) 0.84 V, with a fill factor (FF) of 56% and series resistance (Rs) of 225.43 Ω·cm^2^, resulting in a PCE of 8.1%. After the incorporation of different NS1–NS3 WSe_2_ NSs, the performances of the PSC devices were considerably improved. Further, the observed PSC outcomes are provided in Table 1. After the incorporation of WSe_2_ NSs into the active layer, the light-absorption capacity of the composite films significantly improved compared to the pristine device, which promoted the exciton generation rate. The results reveal that the device with the NS2 (1.5 wt%) WSe_2_ NSs active layer produces the highest J_SC_ of 19.78 mA/cm^2^, V_OC_ of 0.85 V, Rs of 122.02 Ω·cm^2^ and FF of 55%, with a very promising PCE of 9.2%. The presence of WSe_2_ NSs in the ternary blend provides an additional PBDB-T:PCBM:WSe_2_ interface, thus inducing a large interfacial area for charge separation, and it thereby accelerates the rate of exciton dissociation. Moreover, the highly conductive 2D network of NS2 WSe_2_ NSs offers new interconnected percolation networks for charge-carrier transport and collection, which could improve the electron mobility, resulting in larger J_SC_. The observed low Rs further establishes the improved solar cell performances for WSe_2_-incorporating active layers. The observed values are provided with standard deviation (Table 1) between their five replicated experiments to prove their stable performances. Similarly, the J–V characteristics of the constructed devices using different concentrations, such as 1, 1.5 and 2 wt%, of NS2 WSe_2_ blended PBDB-T:PCBM:WSe_2_ in their active layer are provided in Figure 6b. The observed results clearly illustrate the improved behavior of the 1.5 wt% blended active layers. The detailed PSC parameters are presented in the Table 2. In addition, in order to realize the role of concentration variation in achieving high performance, AFM measurements were performed to study the topography of the active layer prepared on ITO-coated glass. Figure S1 shows the 3D AFM topographical images of pure and different concentrations (1, 1.5 and 2 wt%) of the NS2 WSe_2_-doped PBDB-T:PCBM heterojunction active layer. The RMS surface roughness of the active layer is 1.73 nm, 1.59 nm, 1.49 nm and 1.53 nm for the pure and 1, 1.5 and 2 wt% NS2 WSe_2_-doped PBDB-T:PCBM active layers, respectively. The development of a very dense PCDTB-T:PCBM:WSe_2_ NS2 (1.5 wt%) active layer could prevent the leakage of current and produce conduits useful for charge conveyance and separation, improving the device’s performance [56]. Further, to validate the performances of the devices with different concentrations (1, 1.5, and 2 wt%) of NS1 and NS3 WSe_2_ doping, PSC J–V profiles are provided in Figures S2 and S3, respectively.

**Figure 7:**
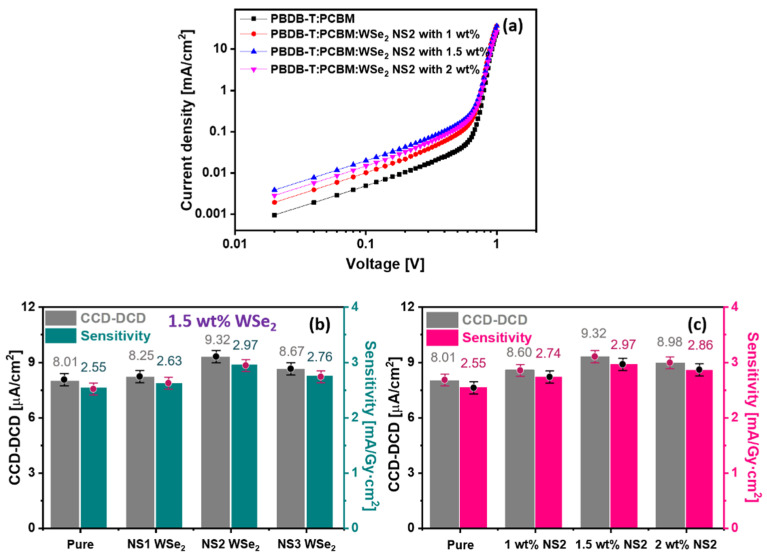
(**a**) Log J–V characteristics and (**b**) CCD-DCD and sensitivity variations (with the standard deviation error bar) Figure S1. NS3 WSe2 NSs blended PBDB-T:PCBM active layers as assessed by X-ray detectors; (**c**) CCD-DCD and sensitivity variations (with the standard deviation error bar) for pure and different concentration of NS2 WSe2 NSs blended PBDB-T:PCBM active layers assessed via X-ray detectors.

The fabricated X-ray detectors were measured using the X-ray generator and electrometer, as described in the experimental part. Figure 7a shows the logarithmic J–V characteristics of pure and NS1–NS3 WSe_2_ NSs (1.5 wt%) blended PBDB-T:PCBM active layers using prepared detectors. For the pristine PBDB-T:PCBM active layer, the X-ray detector achieves a 2.55 mA/Gy·cm^2^ sensitivity. When the active layer is blended with NS1–NS3 WSe_2_ NSs, the sensitivity is increased to 2.63, 2.97, and 2.76 mA/Gy·cm^2^ for NS1, NS2 and NS3, respectively (Figure 7b, right-side axis). The extracted CCD-DCDs (Figure 7b, left side axis) are at 8.01, 8.25, 9.32, and 8.67 μA/cm^2^ for the pure and NS1–NS3 blended PBDB-T:PCBM active layers using the prepared X-ray detectors, respectively. Similarly, the outcomes of different NS2 WSe_2_ NSs blended PBDB-T:PCBM active layers using prepared X-ray detectors are provided in Figure 7c. The sensitivity as assessed by X-ray realizes gives values of 2.55, 2.74, 2.97, and 2.86 mA/Gy·cm^2^ for the pristine PBDB-T:PCBM active layer and 1 wt%, 1.5 wt% and 2 wt% NS2 WSe_2_ NSs, respectively (as shown in Figure 7c, right-side axis). The estimated CCD-DCD values are 8.01, 8.60, 9.32, and 8.98 μA/cm^2^ for the pristine PBDB-T:PCBM active layer and 1 wt%, 1.5 wt% and 2 wt% NS2 WSe_2_ NSs, respectively. The observed X-ray detector outcomes for NS1 and NS3 WSe_2_ NSs (1, 1.5, and 2 wt%) blended PBDB-T:PCBM active layers are provided in Figures S3 and S4, respectively. Better outcomes are ensured for the 1.5 wt% NS2 WSe_2_ NSs hybrid active layer under X-ray detection due to the enhanced conductivity, improved light absorption capacity, and superior mobility.

The carrier mobility was determined using the space-charge-limited-current (SCLC) method in the dark, and it was obtained using the modified Mott–Gurney equation as shown below:

(4) $$\mu =\frac{8}{9}\times J\times \frac{L^{3}}{V_{a}{}^{2}\times \varepsilon_{0}\times \varepsilon_{r}}$$

where *ε*_0_ is the permittivity of free space (=8.85 × 10^−12^ F·m^−1^), *ε_r_* is the relative permittivity of the active layer, *V_a_* is the voltage applied across the detector, *μ* is the carrier mobility, and *L* is the thickness of the active layer. The calculated mobilities using the detector are at 5.62 × 10^−5^, 5.23 × 10^−4^, 8.82 × 10^−4^, and 7.13 × 10^−4^ cm^2^/V·s for the pristine active layer and 1 wt%, 1.5 wt% and 2 wt% of NS2 WSe_2_ NSs blended active layers, respectively.

## 4. Conclusions

In this work, we have ultrasonically prepared exfoliated WSe_2_ NSs and blended them with PBDB-T:PCBM as an active layer for ternary hybrid solar cells and X-ray detectors. The different types (NS1–NS3) of WSe_2_ NSs were incorporated with active layers to explore their potentials to alter the electron transport behavior in the prepared devices. The TMD WSe_2_ blended active layer produced a synergistic enhancement of exciton generation and dissociation, and enhanced hole and electron transport through the active layer, which helped in achieving the high J_SC_ and PCE for solar cells and the high sensitivity of detectors. The highest PCE of 9.2% was attained for the NS2 WSe_2_ (1.5 wt%) blended PBDB-T:PCBM active layer, which is higher than the devices comprised of pristine and NS1 and NS3 blended active layers. The fabricated X-ray detectors achieved the maximum CCD-DCD of 9.32 μA/cm^2^, a high sensitivity of 2.97 mA/Gy·cm^2^, and large carrier mobility of 8.82 × 10^−4^ cm^2^/V·s for the NS2 WSe_2_ (1.5 wt%) blended PBDB-T:PCBM active layer. Our research work provides a good strategy for incorporating highly dispersed aggregated WSe_2_ NSs in an active layer to promote the charge extraction process and electron/hole transport behavior, thus realizing high-performance semiconductor devices. These results offer a new direction for the development of high-performance devices based on hybrid structures for future electronics.

## Figures and Tables

**Table 1:** PSC performances of pristine and different WSe_2_ NSs blended PBDB-T:PCBM active layers using constructed devices (± indicates the standard deviation).

WSe_2_ Type	Doping wt%	Voc (V)	J_SC_ (mA/cm^2^)	FF (%)	PCE (%)	Rs (Ω·cm^2^)
-	0 (Pure)	0.84 ± 0.01	16.81 ± 0.13	56 ± 1	8.1 ± 0.09	225.43 ± 2.78
NS1	1.5	0.84 ± 0.01	18.14 ± 0.17	54 ± 1	8.4 ± 0.14	144.38 ± 3.15
NS2	1.5	0.85 ± 0.01	19.78 ± 0.19	55 ± 1	9.2 ± 0.17	122.02 ± 4.76
NS3	1.5	0.85 ± 0.01	18.56 ± 0.18	55 ± 1	8.7 ± 0.15	136.81 ± 3.59

**Table 2:** PSC performances of pristine and different amounts NS2 WSe_2_ NSs blended into the PBDB-T:PCBM active layer using constructed devices (± indicates the standard deviation).

NS2 WSe_2_ (wt%)	Voc (V)	J_SC_ (mA/cm^2^)	FF (%)	PCE (%)	Rs (Ω·cm^2^)
0 (Pure)	0.84 ± 0.01	16.81 ± 0.13	56 ± 1	8.1 ± 0.09	225.43 ± 2.78
1	0.85 ± 0.01	18.27 ± 0.17	55 ± 1	8.6 ± 0.15	157.33 ± 3.42
1.5	0.85 ± 0.01	19.78 ± 0.19	55 ± 1	9.2 ± 0.17	122.02 ± 4.76
2	0.85 ± 0.01	18.69 ± 0.18	56 ± 1	8.9 ± 0.16	126.13 ± 3.85

## Data Availability

Data Sharing is not applicable.

## Supplementary Materials

The following are available online at https://www.mdpi.com/article/10.3390/ma14123206/s1, Table S1: Ultrasonic preparation parameters for WSe_2_ nanosheets. Table S2: Active layer preparation parameters. Figure S1. Atomic force measurement (AFM) image (a) PBDB-T:PCBM, (b) PBDB-T:PCBM:WSe_2_NS2 with 1 wt%, (c) PBDB-T:PCBM:WSe_2_ NS2 with 1.5 wt% and (d) PBDB-T:PCBM:WSe_2_ NS2 with 2 wt%, Figure S2: J–V characteristics and their outcomes for pristine and different amounts NS1 WSe_2_ NSs blended PBDB-T:PCBM active layer, Figure S3: J–V characteristics and their outcomes for pristine and different amounts NS3 WSe_2_ NSs blended PBDB-T:PCBM active layer, Figure S4: CCD-DCD and sensitivity variations for pure and different concentrations of NS1 WSe_2_ NSs blended PBDB-T:PCBM active layer comprised X-ray detectors, Figure S5: CCD-DCD and sensitivity variations for pure and different concentrations of NS3 WSe_2_ NSs blended PBDB-T:PCBM active layer comprised X-ray detectors, Table S3: Photovoltaic parameters of polymer/2D materials–based PSCs.
