# Supplementary material for: Ultrasonically Processed WSe2 Nanosheets Blended Bulk Heterojunction Active Layer for High-Performance Polymer Solar Cells and X-ray Detectors
Source: Materials (Basel). 2021 Jun 10;14(12):3206. doi: 10.3390/ma14123206 (PMC8230459; doi:10.3390/ma14123206)
Supplement: Supplementary file 1 [file materials-14-03206-s001.zip › materials-1220139-SI.pdf]

materials

MDPI

Article

Ultrasonically Processed WSe_2 Nanosheets Blended Bulk Heterojunction Active Layer for High-Performance Polymer Solar Cells and X-ray Detectors

Hailiang Liu [1,†], Sajjad Hussain [2,†], Jehoon Lee [1], Dhanasekaran Vikraman [3,*] and Jungwon Kang [1,*]

1 Department of Electronics and Electrical Engineering, Dankook University, Gyeonggi-do 16890, Korea; liuhailiang107@gmail.com (H.L.); usyj0512@gmail.com (J.L.)
2 Institute of Nano and Advanced Materials Engineering, Sejong University, Seoul 143-747, Korea; shussainawan@gmail.com
3 Division of Electronics and Electrical Engineering, Dongguk University-Seoul, Seoul 04620, Korea
* Correspondence: v.j.dhanasekaran@gmail.com (D.V.); jkang@dankook.ac.kr (J.K.)
† Authors contributed equally.

Citation: Liu, H.; Hussain, S.; Lee, J.; Vikraman, D.; Kang, J. Ultrasonically-Processed WSe_2 Nanosheets Blended Bulk Heterojunction Active Layer for High-Performance Polymer Solar Cells and X-ray Detectors. *Materials* **2021**, *14*, 3206. https://doi.org/10.3390/ma14123206

Academic Editor: Marko Topic

Received: 28 April 2021
Accepted: 7 June 2021
Published: 10 June 2021

Publisher's Note: MDPI stays neutral with regard to jurisdictional claims in published maps and institutional affiliations.

Copyright: © 2021 by the authors. Licensee MDPI, Basel, Switzerland. This article is an open access article distributed under the terms and conditions of the Creative Commons Attribution (CC BY) license (http://creativecommons.org/licenses/by/4.0/).

Table S1. Ultrasonic preparation parameters for WSe_2 nanosheets.

Sample Code	Sonication Time (h)	Ultrasonic Power (W)
NS1	6	60
NS2	12	60
NS3	18	60

Table S2. Active layer preparation parameters.

Spin Coating	Acceptor/Donor Layer Preparation			WSe_2 Doping Material		
	Material	Ratio	Solvent	Type	Solvent	Doping Concentration (wt%)
1100 rpm / 30 s	PBDB-T:PCBM	2:3	chlorobenzene	NS1	isopropyl alcohol	0
						1
						1.5
						2
1100 rpm / 30 s	PBDB-T:PCBM	2:3	chlorobenzene	NS2	isopropyl alcohol	0<br>1<br>1.5<br>2
1100 rpm / 30 s	PBDB-T:PCBM	2:3	chlorobenzene	NS3	isopropyl alcohol	0<br>1<br>1.5<br>2

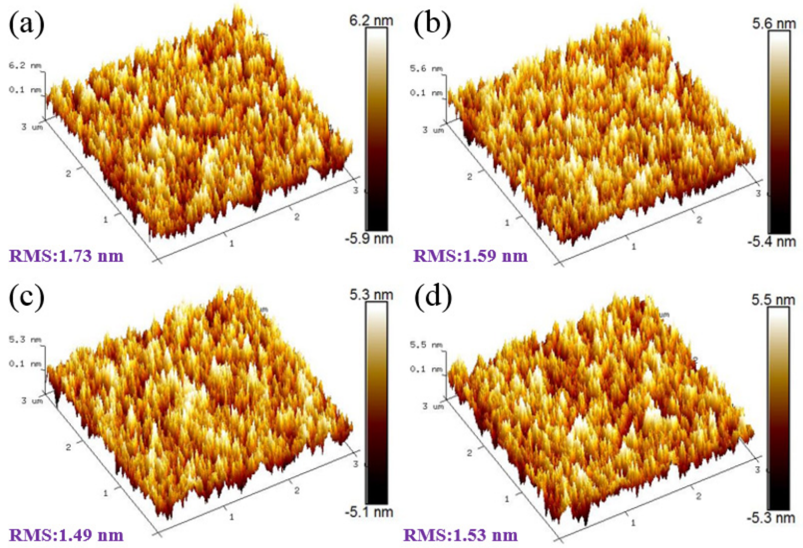

Figure S1. Atomic force measurement (AFM) image (**a**) PBDB-T:PCBM, (**b**) PBDB-T:PCBM:WSe_2NS2 with 1 wt%, (**c**) PBDB-T:PCBM:WSe_2 NS2 with 1.5 wt% and (**d**) PBDB-T:PCBM:WSe_2 NS2 with 2 wt%.

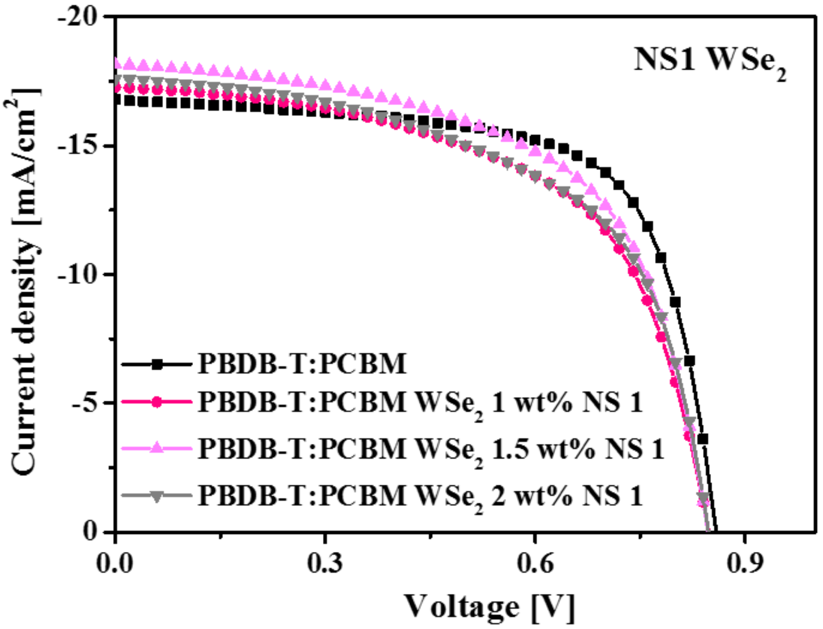

NS1 WSe_2 (wt%)	Voc (V)	J_{SC} (mA/cm^2)	FF (%)	PCE (%)	Rs ($\Omega\cdot cm^2$)
0 (Pure)	0.84 ± 0.01	16.81 ± 0.13	56 ± 1	8.1 ± 0.09	225.43 ± 2.78
1	0.85 ± 0.01	17.27 ± 0.14	55 ± 1	8.2 ± 0.11	198.87 ± 2.89
1.5	0.84 ± 0.01	18.14 ± 0.17	54 ± 1	8.4 ± 0.14	144.38 ± 3.15
2	0.85 ± 0.01	17.58 ± 0.15	55 ± 1	8.3 ± 0.13	156.75 ± 3.08

Figure S2. J–V characteristics and their outcomes for pristine and different amounts NS1 WSe_2 NSs blended PBDB-T:PCBM active layer.

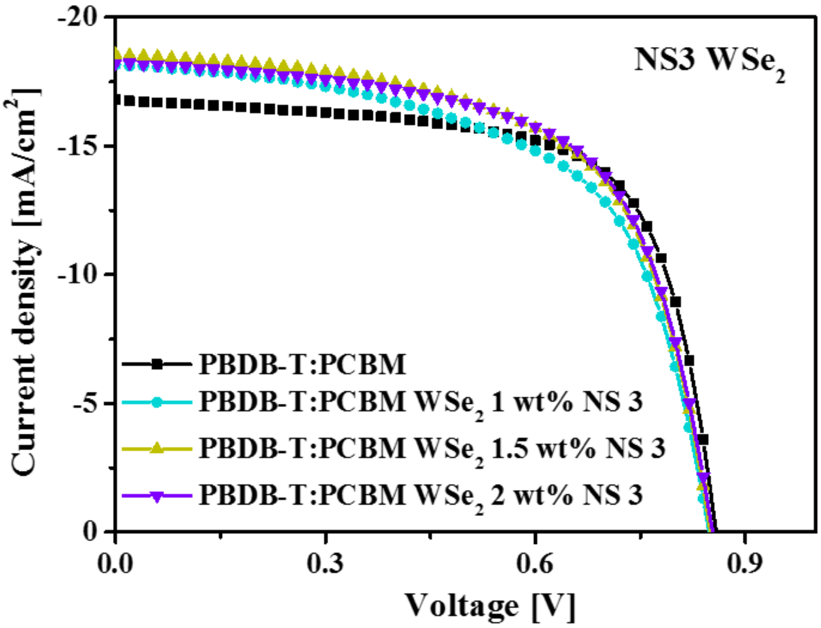

NS3 WSe_2 (wt%)	Voc (V)	Jsc (mA/cm²)	FF (%)	PCE (%)	Rs (Ω·cm²)
0 (Pure)	0.84 ± 0.01	16.81 ± 0.13	56 ± 1	8.1 ± 0.09	225.43 ± 2.78
1	0.85 ± 0.01	18.17 ± 0.15	54 ± 1	8.4 ± 0.11	151.28 ± 3.12
1.5	0.85 ± 0.01	18.56 ± 0.18	55 ± 1	8.7 ± 0.15	136.81 ± 3.59
2	0.85 ± 0.01	18.22 ± 0.16	55 ± 1	8.5 ± 0.13	149.66 ± 3.41

Figure S3. J–V characteristics and their outcomes for pristine and different amounts NS3 WSe_2 NSs blended PBDB-T:PCBM active layer.

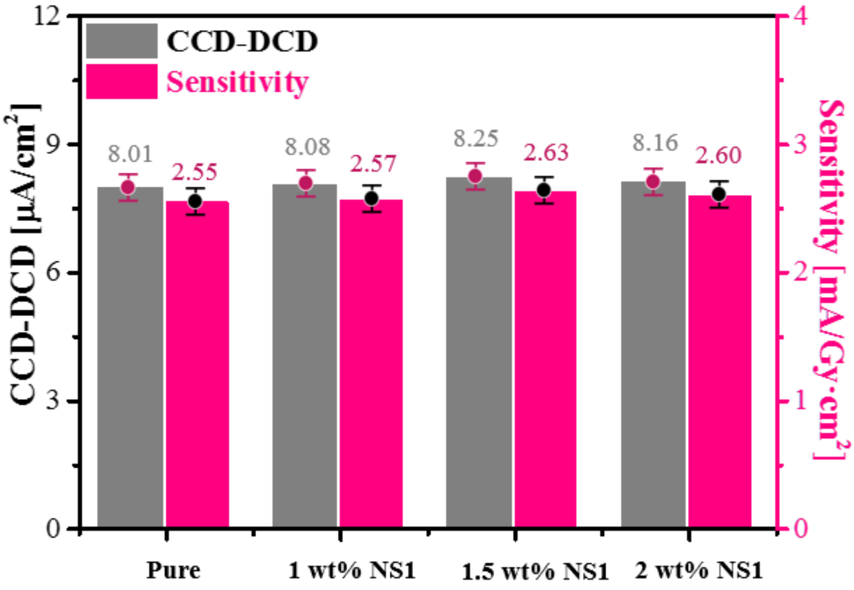

Figure S4. CCD-DCD and sensitivity variations for pure and different concentrations of NS1 WSe_2 NSs blended PBDB-T:PCBM active layer comprised X-ray detectors.

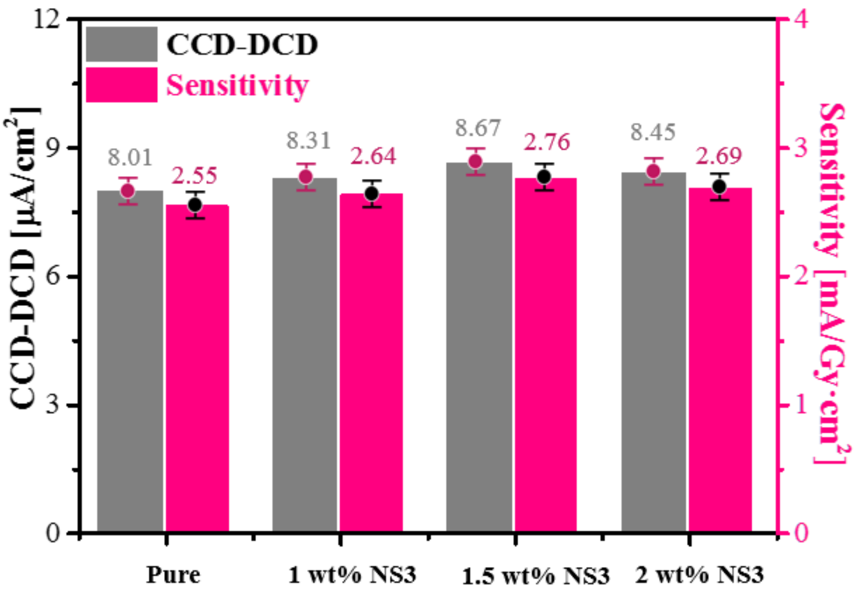

Figure S5. CCD-DCD and sensitivity variations for pure and different concentrations of NS3 WSe_2 NSs blended PBDB-T:PCBM active layer comprised X-ray detectors.

Table S3. Photovoltaic parameters of polymer/2D materials–based PSCs.

Donor	Acceptor	Doping Material	Doping Ratio (%)	PCE (%)	Increase Range (%)	Reference
PBDB-T	PCBM	WSe_2 NS2	0	8.1	13.5	This work
			1.5	9.2		
PTB7	PCBM	WSe_2 (Sample 1)	0	8.22	4.25	[1]
			2	8.57		
PTB7	PCBM	WSe_2 (Sample 2)	0	8.22	14.96	
			2	9.45		
PTB7	PCBM	WSe_2 (Sample 3)	0	8.22	8.39	
			2	8.91		
PTB7	PCBM	GOQD	0	6.70	6.11	[2]
			0.2	7.11		
PTB7	PCBM	GQD 5	0	6.70	13.43	
			0.5	7.60		
PTB7	PCBM	GQD 10	0	6.70	7.61	
			0.02	7.21		
PCDTBT	PCBM	WS_2	0	5.6	8.92	[3]
			1.5	6.1		
PCDTBT	PCBM	WS_2-Au	0	5.6	12.5	
			1.5	6.3		
P3HT	PCBM	Graphene	0	2.79	13.62	[4]
			2	3.17		
PCDTBT	PCBM	rGO-Sb_2S_3	0	5.58	25.44	[5]
			0.25	7.00		
P3HT	PCBM	N-rGO	0	3.19	40.43	[6]
			0.5	4.48		

References

1. Kakavelakis, G.; Castillo, A.E.D.R.; Pellegrini, V.; Ansaldo, A.; Tzourmpakis, P.; Brescia, R.; Prato, M.; Stratakis, E.; Kymakis, E.; Bonaccorso, F. Size-Tuning of WSe2 Flakes for High Efficiency Inverted Organic Solar Cells. *ACS Nano* **2017**, *11*, 3517–3531, doi:10.1021/acsnano.7b00323.
2. Kim, J.K.; Park, M.J.; Kim, S.J.; Wang, N.H.; Cho, S.P.; Bae, S.; Park, J.H.; Hong, B.H. Balancing Light Absorptivity and Carrier Conductivity of Graphene Quantum Dots for High-Efficiency Bulk Heterojunction Solar Cells. *ACS Nano* **2013**, *7*, 7207–7212, doi:10.1021/nn402606v.
3. Sygletou, M.; Tzourmpakis, P.; Petridis, C.; Konios, D.; Fotakis, C.; Kymakis, E.; Stratakis, E. Laser induced nucleation of plasmonic nanoparticles on two-dimensional nanosheets for organic photovoltaics. *J. Mater. Chem. A* **2016**, *4*, 1020–1027, doi:10.1039/c5ta09199c.
4. Robaeys, P.; Bonaccorso, F.; Bourgeois, E.; D'Haen, J.; Dierckx, W.; Dexters, W.; Spoltore, D.; Drijkoningen, J.; Liesenborgs, J.; Lombardo, A.; et al. Enhanced performance of polymer:fullerene bulk heterojunction solar cells upon graphene addition. *Appl. Phys. Lett.* **2014**, *105*, 083306, doi:10.1063/1.4893777.
5. Balis, N.; Konios, D.; Stratakis, E.; Kymakis, E. Ternary Organic Solar Cells with Reduced Graphene Oxide-Sb2S3Hybrid Nanosheets as the Cascade Material. *ChemNanoMat* **2015**, *1*, 346–352, doi:10.1002/cnma.201500044.
6. Jun, G.H.; Jin, S.H.; Lee, B.; Kim, B.H.; Chae, W.-S.; Hong, S.H.; Jeon, S. Enhanced conduction and charge-selectivity by N-doped graphene flakes in the active layer of bulk-heterojunction organic solar cells. *Energy Environ. Sci.* **2013**, *6*, 3000–3006, doi:10.1039/c3ee40963e.
